# Identification of neoantigens and immunological subtypes in clear cell renal cell carcinoma for mRNA vaccine development and patient selection

**DOI:** 10.18632/aging.204798

**Published:** 2023-06-13

**Authors:** Daoqi Zhu, Jiabin Yang, Minyi Zhang, Zhongxiao Han, Meng Shao, Qin Fan, Yun Ma, Dandan Xie, Wei Xiao

**Affiliations:** 1 School of Traditional Chinese Medicine, Southern Medical University, Guangzhou 510515, Guangdong, China; 2 Department of Epidemiology, School of Public Health, Southern Medical University, Guangzhou 510515, Guangdong, China; 3 Guangdong Provincial Key Laboratory of Chinese Medicine Pharmaceutics, School of Traditional Chinese Medicine, Southern Medical University, Guangzhou 510515, Guangdong, China; 4 Department of pharmacy, Nanfang Hospital, Southern Medical University, Guangzhou 510515, Guangdong, China; 5 The Affiliated TCM Hospital of Guangzhou Medical University, Guangzhou 510130, Guangdong, China

**Keywords:** ccRCC, mRNA vaccine, tumor antigens, immune cancer subtypes, cancer therapy

## Abstract

Clear cell renal cell carcinoma (ccRCC) is a common urological malignancy with diverse histological types. This study aimed to detect neoantigens in ccRCC to develop mRNA vaccines and distinguish between ccRCC immunological subtypes for construction of an immune landscape to select patients suitable for vaccination. Using The Cancer Genome Atlas SpliceSeq database, The Cancer Genome Atlas, and the International Cancer Genome Consortium cohorts, we comprehensively analysed potential tumour antigens of ccRCC associated with aberrant alternative splicing, somatic mutation, nonsense-mediated mRNA decay factors, antigen-presenting cells, and overall survival. Immune subtypes (C1/C2) and nine immune gene modules of ccRCC were identified by consistency clustering and weighted correlation network analysis. The immune landscape as well as molecular and cellular characteristics of immunotypes were assessed. Rho-guanine nucleotide exchange factor 3 (ARHGEF3) was identified as a new ccRCC antigen for development of an mRNA vaccine. A higher tumour mutation burden, differential expression of immune checkpoints, and immunogenic cell death were observed in cases with the C2 immunotype. Cellular characteristics increased the complexity of the immune environment, and worse outcomes were observed in ccRCC cases with the C2 immunotype. We constructed the immune landscape for selecting patients with the C2 immunotype suitable for vaccination.

## INTRODUCTION

Renal cell carcinoma (RCC) is among the top 10 cancers worldwide. It is a commonly diagnosed and histologically diverse urologic malignancy accounting for >90% of all renal neoplasms, with clear cell RCC (ccRCC) representing almost 75% of cases [1]. Localized RCC is treatable surgically by either radical or partial nephrectomy; nevertheless, ∼30% of localized ccRCC metastasize at some point [2]. RCC major risk factors include obesity, hypertension, and smoking, and the incidence increases markedly with age. The pathogenesis of ccCCR is closely related to several genetic mutations, including *VHL*, [3] *PBRM1*, [4] *SETD2*, [4] *BAP1*, [5] *KDM5C*, [4] and *mTOR* mutations [6]. However, ccRCC development requires supplementary epigenetic and genetic events [7]. As RCCs are highly vascular, it is not surprising that tyrosine kinase inhibitors, [8] anti-VEGF monoclonal antibodies, [9] and mTOR [10] inhibitors have been used to explore their features. Unfortunately, there are no available clinical markers to classify patients for therapies, despite intensive efforts. With respect to immunotherapy, nivolumab response rate is only 25%, and there was no significant decrease in tumor size in most patients treated with nivolumab [11]. The relevance of the International Metastatic Renal Cell Carcinoma Database Consortium (IMDC) prognostic criteria remains to be established in the era of frontline combination immunotherapy [12]. In the absence of prognostic standards based on alternative immunotherapy, the IMDC criteria continue to be used in clinical trials to stratify patients at risk under the guide of clinical guidelines. Immune checkpoint (ICP) blockade (ICB) therapy increases the overall survival (OS) rates of advanced RCC cases treated with nivolumab [11].

Tumor mutation burden (TMB) is a clinically related parameter that highlights the molecular characteristics associated with immunotherapy responses [13]. Splicing of pre-mRNA is significant to the pathology of various diseases, especially cancer. Aberrant splicing isoforms are highlighted as tumor markers and cancer therapy targets [14]. Aberrantly expressed transcripts in cancer cells are degraded by nonsense-mediated mRNA decay (NMD), a process involved in the mRNA quality control system [15]. NMD disruption factors have significant correlation with the splicing isoform count in TCGA-Lung adenocarcinoma datasets; [16] the precise identification of aberrant transcripts in cancer cells is essential for identifying potential neoantigens. Particularly, the initial hypothesis based on TMB as an immunotherapy-relevant parameter is associated with the fact that somatic variants can generate tumor-specific neoantigens. Several clinical trials have used TMB as an important stratification factor or a landmark endpoint to decipher the role of TMB in cancer-type treatment decision-making [17]. A minority of somatic mutations in DNA can give rise to neoantigens, but not all neopeptides present on the cell surface are immunogenic [18]. Consequently, TMB analysis to detect the underlying mutations subsets responsible for immunogenicity may enable substantial optimization of biomarker accuracy and improved therapeutic targeting of neoantigens [19]. Hence, profiling abnormal transcripts is equally essential as a potential biomarker of an ICP inhibitor (ICI).

Although mRNA-based cancer vaccines have been extensively reviewed, with the recent approval of two mRNA lipid nanoparticle vaccines for coronavirus disease 2019 (COVID-19), the focus has again shifted to mRNA vaccines as a promising platform for cancer immunotherapy. Recently, the Massachusetts Institute of Technology (MIT) Technology Review released the 2021 list of “Top 10 Breakthrough Technologies in the World,” and mRNA vaccines that have caused significant changes in the field of medicine topped the list. MIT commented that mRNA technology has broad application prospects for treating various infectious diseases (including COVID-19 and malaria) and cancer, among other diseases. mRNA vaccines are rapidly advancing in preclinical and clinical studies of cancer and infectious diseases [20]. We can design cancer vaccines to target tumor-associated unique antigens that have preferential expression in cancerous cells, and to achieve this goal, multiple preclinical and clinical trials of mRNA vaccines have been conducted [21]. Compared to an infectious disease vaccine, the challenge in developing a cancer vaccine lies in the limitation of clinical translation because of the difficulty in predicting antigens and their poor immunogenicity. Tumor antigens vary significantly among cancer patients; hence, identifying immunogenic tumor-associated antigens and tumor-specific antigens and overcoming the inhibitory tumor microenvironment remain significant obstacles for the development of mRNA-based cancer vaccines. Here, we aimed to identify novel ccRCC antigens and different immune subtypes for developing mRNA-based cancer vaccines and mapping the immune landscape of ccRCC to select patients fitting the criteria for vaccination.

## RESULTS

### Identification of aberrant alternative splicing (AS) events and potential ccRCC antigens

**Figure 1:**
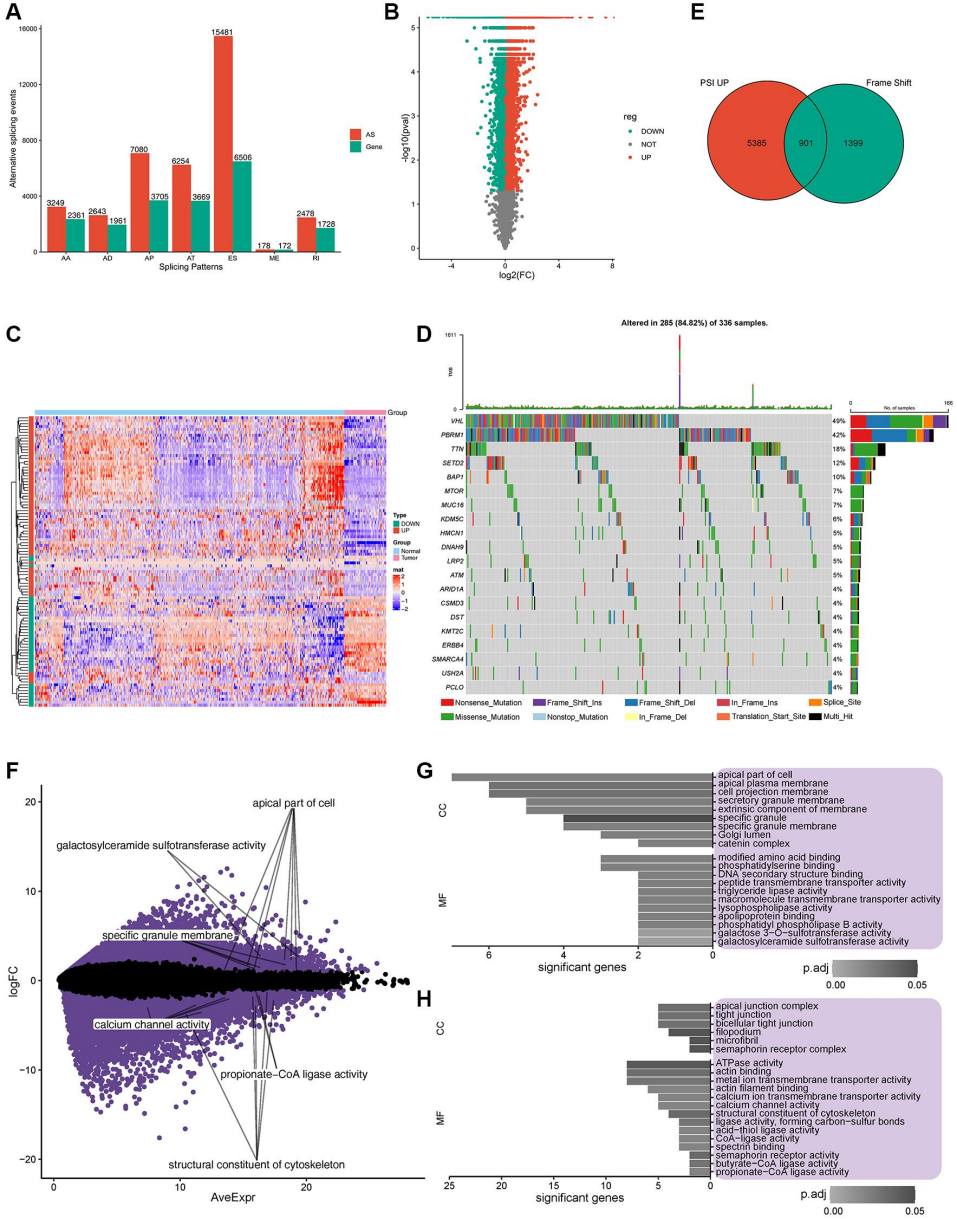
**Potential antigen identification and enrichment of gene ontology (GO).** (**A**) Overview of alternate splicing (AS) in ccRCC patients. (**B**) PSI of all the AS events, Green represents downregulated gene expression, while red represents upregulated gene expression. (**C**) Heatmap of PSI for the top 100 AS events. (**D**) Twenty highly mutated genes in ccRCC patients from TCGA cohort. (**E**) Total of 901 potential antigens were found in PSI, which were significantly upregulated after frame shift mutations, Green represents frame shift genes, while red represents PSI up genes. (**F**) Expression of 156 potential antigens was significantly regulated. (**G**, **H**). Frequency of somatic mutations and genes/patients involved. (**G**, **H**). GO enrichment of significantly upregulated/downregulated potential antigens.

To identify the features of aberrant AS in ccRCC patients, seven AS events compared with normal samples were screened from TCGA cohort (Supplementary Figure 1A); exon skip (ES) had the highest incidence (15481), appearing in 6506 genes, followed by the alternative promoter (AP) event. In contrast, the lowest mutually exclusive exons (MEs) occurred 178 times in 172 genes (Figure 1A; Supplementary Figure 1B). A PSI value can be used to evaluate the variation of AS events; the PSI values for 11289 and 12049 AS events increased and decreased, respectively, in ccRCC patients (Figure 1B). The heatmap of PSI for the top 100 AS events is shown in Figure 1C. Gene mutations, especially splice site mutations, may lead to aberrant splicing isoforms; [22] consequently, we analyzed and visualized somatic mutations (MuTect2 variant aggregation and masking) in ccRCC patients from TCGA cohort (Figure 1D). Among the entire cohort of patients included in the database, it was observed that 49% of the patients presented with VHL gene mutations, while 42% had mutations in the PBRM1 gene [4, 23]. Importantly, within the subset of patients exhibiting mutations in either the VHL or PBRM1 gene, approximately half of them concurrently possessed mutations in both genes. Missense mutations occurred most frequently in 5689 genes, followed by frameshift deletion in 578 genes (Supplementary Figure 1C). Multiple mutations commonly occurred in one sample, and the top three mutation types were missense mutations, frameshift deletions, and nonsense mutations (Supplementary Figure 1D). The distributions of the top 10 mutated genes in all individuals and ccRCC patients are shown in Supplementary Figure 1E, 1F, respectively. Our data revealed that missense mutations and frameshift mutations occurred more frequently in patients with ccRCC.

In total, 901 genes were identified as encoding for potential antigens in the PSI significantly upregulated group (including frameshift deletion and frameshift insertion) (Figure 1E). GO enrichment analysis of the 901 potential antigens in terms of biological processes (BP), molecular function (MF), cellular component (CC) is shown in Supplementary Figure 1G–1I. Further analysis of the expression profiles of TCGA cohort revealed 10393 and 8134 genes with upregulated and downregulated expression, respectively. Among these significantly regulated genes (Figure 1F), GO enrichment analysis indicated that potential upregulated antigens were related to the apical part of the cell, apical plasma membrane, and cell projection membrane in CC and modified amino acid binding and phosphatidylserine binding in MF (Figure 1G). Potential downregulated antigens were related to apical junction complex, tight junction, and bicellular tight junction in CC, and ATPase activity, actin binding, and metal ion transmembrane transporter activity in MF (Figure 1H). Furthermore, 156 candidate antigens showed upregulated aberrant AS events with frameshift mutations and abnormal expression.

### Potential antigens related to NMD

**Figure 2:**
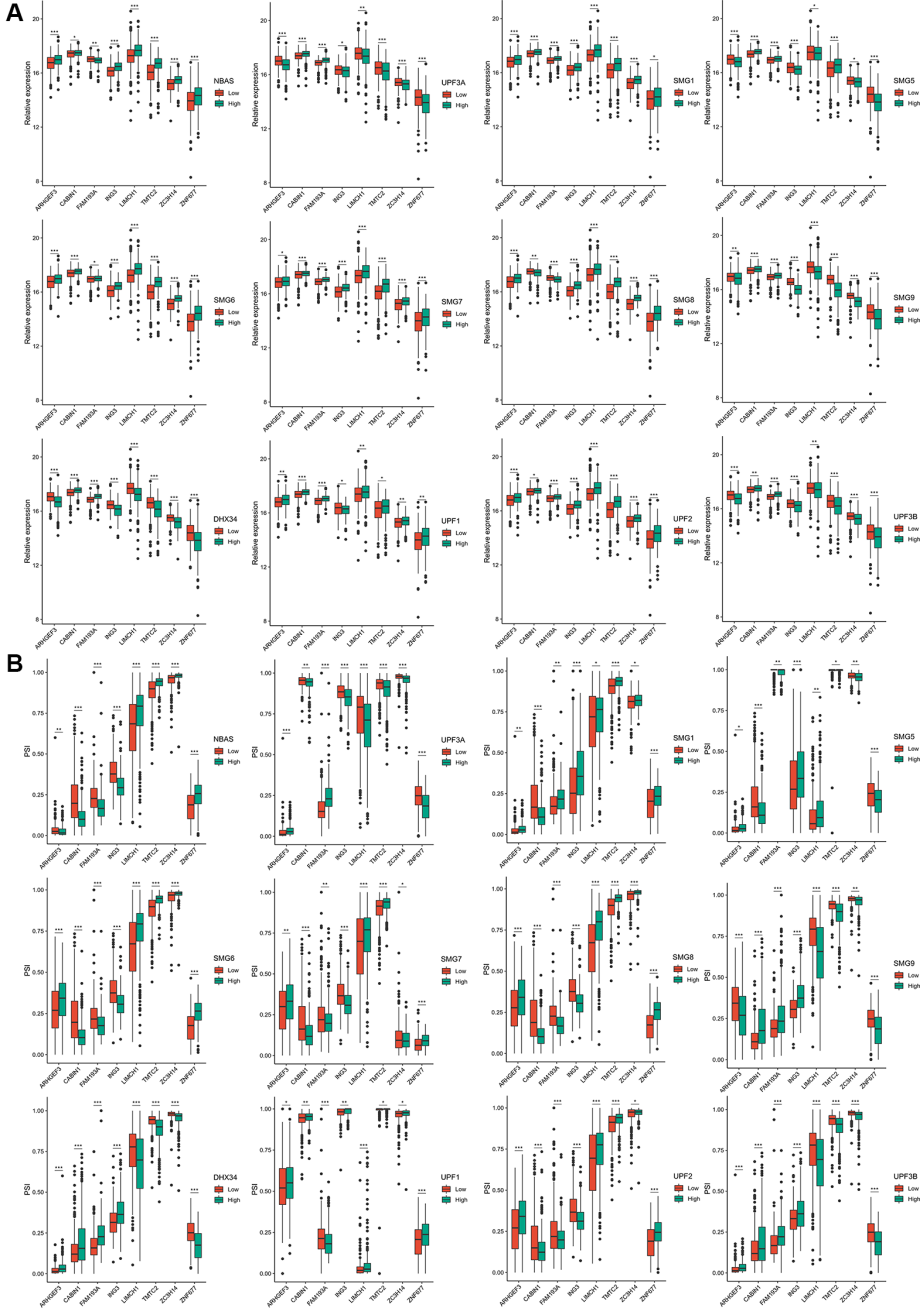
**The expression and PSI of eight potential antigens related to 12 NMD factors.** (**A**) The expression of eight potential antigens between NBAS, UPF3A, SMG1, SMG5, SMG6, SMG7, SMG8, SMG9, DHX34, UPF1, UPF2, UPF3B high-expression and low-expression groups. (**B**) The PSI of eight potential antigens between NBAS, UPF3A, SMG1, SMG5, SMG6, SMG7, SMG8, SMG9, DHX34, UPF1, UPF2, UPF3B high-expression and low-expression groups. ^*^padj < 0.05; ^**^padj < 0.01; ^***^padj < 0.001.

Twelve NMD factors were used to identify the potential antigens in ccRCC. The expression profiles of TCGA datasets were grouped according to the median expression level of each NMD factor. Forty-seven differentially expressed genes were identified with respect to the expression of 12 NMD factors. Notably, the expression of *ARHGEF3*, *CABIN1*, *FAM193A*, *ING3*, *LIMCH1*, *TMTC2*, *ZC3H14*, and *ZNF677* (the low- and high-expression groups) was significantly different among the 12 NMD factors (Figure 2A). Furthermore, 157 differentially expressed PSI genes were observed among the 12 NMD factors (Supplementary Table 1). Similarly, the PSI of *ARHGEF3*, *CABIN1*, *FAM193A*, *ING3*, *LIMCH1*, *TMTC2*, *ZC3H14*, and *ZNF677* was significantly different among the 12 NMD factors in the low- and high-expression groups (Figure 2B).

### Potential antigens related to prognosis and antigen presenting cells (APCs) in ccRCC patients

Cox regression modeling was performed on the potential antigens and OS or DFS data. A total of 510 genes were closely related to OS, whereas 40 genes were closely related to DFS. Twenty-one of these genes were common in both, and 15 potential genes were notably relevant to the survival rate. *PRPF39*, *SPG7*, *PISD*, *TUBGCP6*, *RBM6*, *SORBS2*, *PAM*, *ZFAT*, *DOCK7*, *ZNF266*, *RBM39*, *DFNA5*, *ERMAP*, *BTF3*, *GUSB* were significantly associated with OS (Supplementary Figure 2). Patients overexpressing *PRPF39, SPG7, PISD, TUBGCP6, RBM6, ZNF266, RBM39, DFNA5,* and *GUSB* in the tumor tissues had significantly shorter survival duration than patients in the low-expression group. In contrast, patients with insufficient expression of *SORBS2, PAM, ZFAT, DOCK7, ERMAP, BTF3*, and *GUSB* in the tumor tissues had shorter survival duration than patients in the high-expression group.

**Figure 3:**
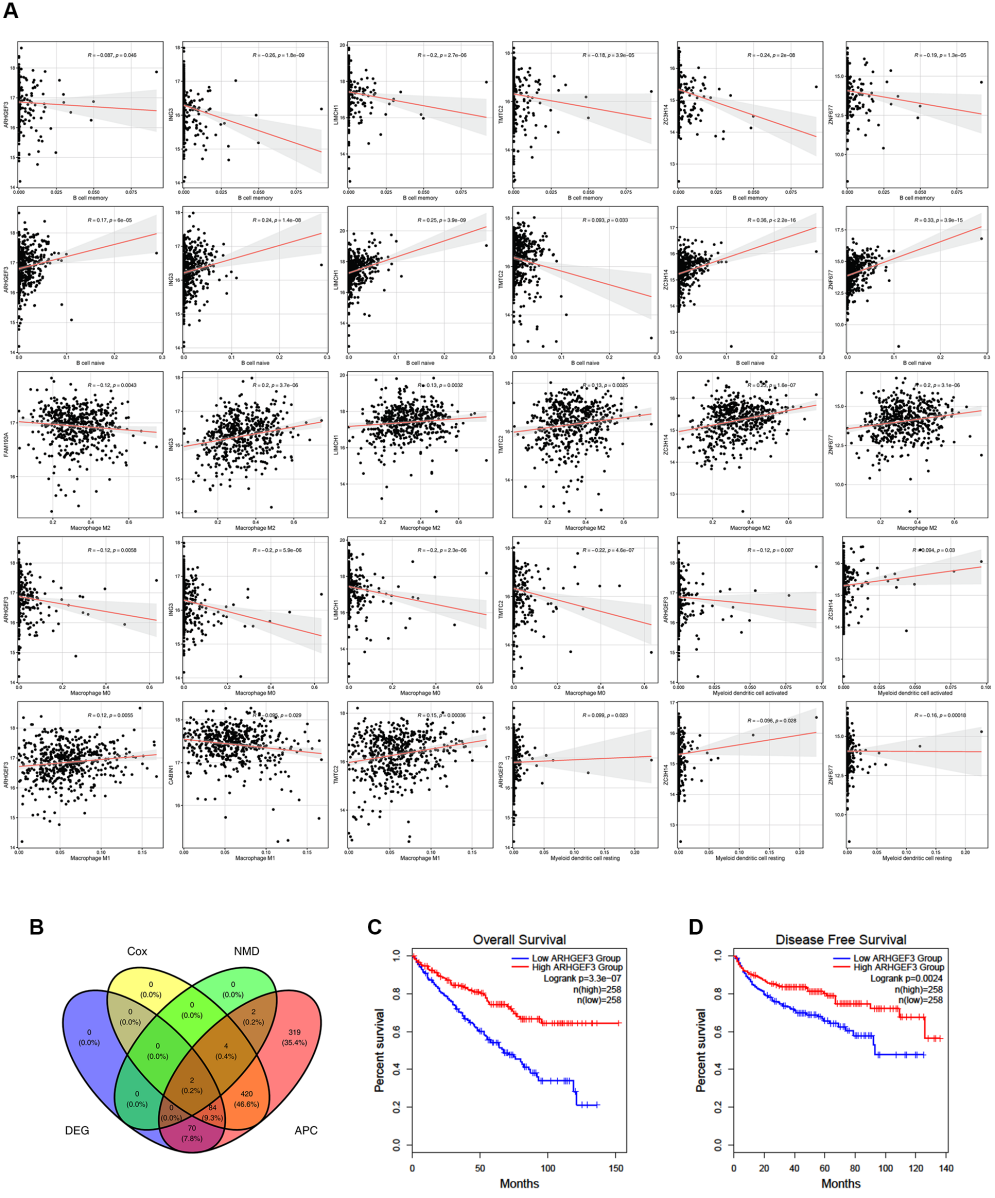
**Potential antigens related to prognosis and antigen presenting cells (APC) in clear ccRCC patients.** (**A**) ARHGEF3, ING3, LIMCH1, TMTC2, ZC3H14, and ZNF677 were closely related to APCs. (**B**) ARHGEF3 and ZNF677 were closely related to the expression difference, NMD factor difference, OS difference, and APC difference. (**C**) Effect of ARHGEF3 on overall survival rate in ccRCC patients. (**D**) Effect of ARHGEF3 on disease free survival rate in ccRCC patients.

Furthermore, six candidate potential antigens related to NMD factors, including, *ARHGEF3*, *ING3*, *LIMCH1*, *TMTC2*, *ZC3H14*, and *ZNF677*, were closely related to APCs (Figure 3A). For example, *ARHGEF3* overexpression was notably associated with decreased infiltration of memory B cells, M0 macrophages, and activated myeloid dendritic cells in tumors; in contrast, infiltration of naïve B cells, macrophage M1 cells, and resting myeloid dendritic cells was increased. *ZNF677* overexpression was associated with decreased infiltration of memory B cells and resting myeloid dendritic cells, but increased infiltration of naïve B cells and M2 macrophages in tumors. These results suggest that we can directly process tumor antigens and present them by APCs to T cells and for recognition by B cells to elicit an immune response. These antigens hold promise as candidates for developing mRNA-based vaccines against ccRCC. Combining the aforementioned results, *ARHGEF3* and *ZNF677* were closely related to differences in the expression of NMD factors, OS, and APC function (Figure 3B–3D). According to TCIA database, ARHGEF3 is the best candidate antigen for mRNA-based vaccine for ccRCC.

### Potential immune subtype identification in ccRCC patients

**Figure 4:**
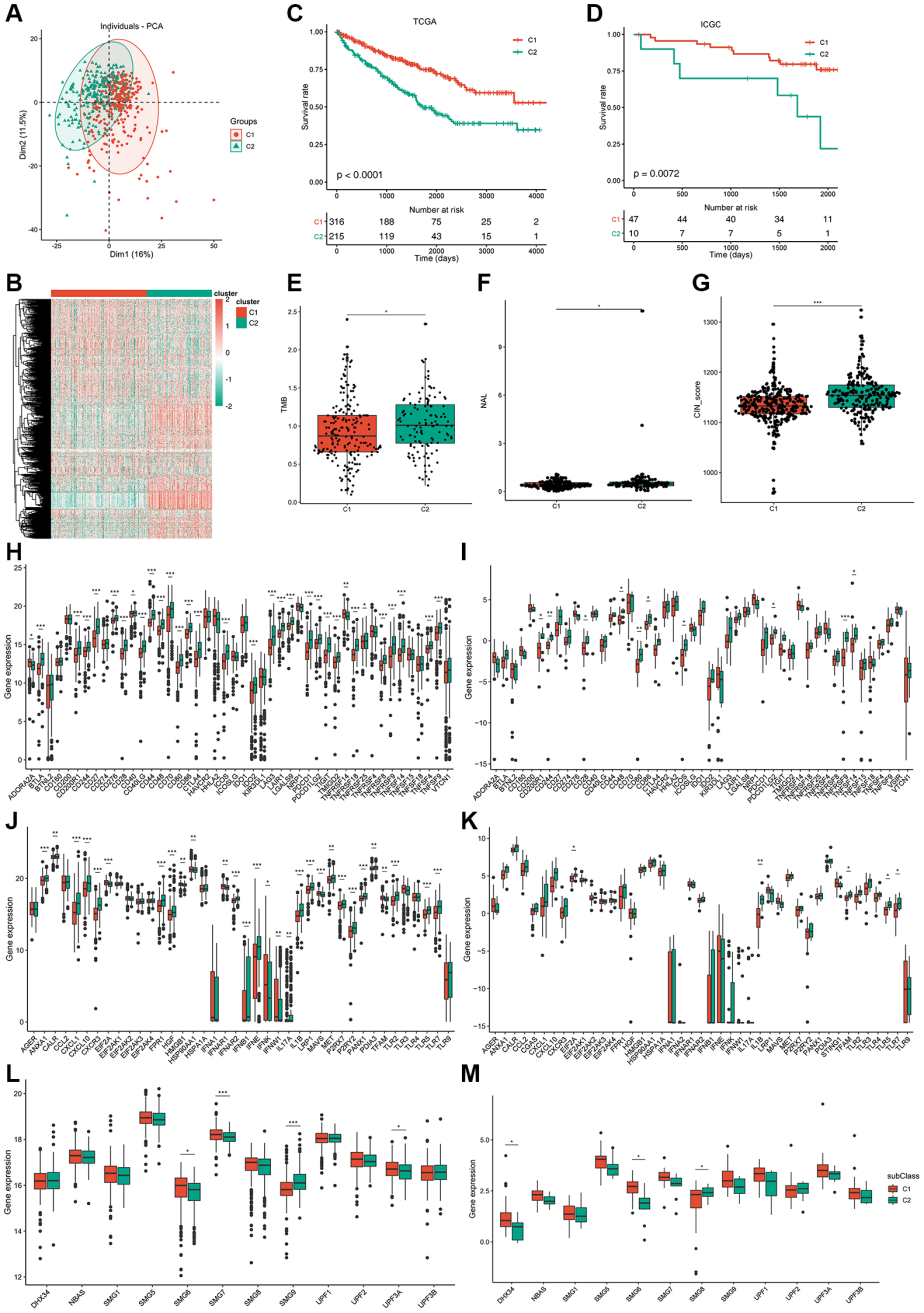
**Identification of potential immune subtypes of ccRCC patients and molecular characteristics.** (**A**) PCA of ccRCC patients in TCGA cohort. (**B**) Heatmap of immune-related genes of C1/C2 ccRCC patients. (**C**, **D**) Kaplan-Meier curves showing overall survival (OS) of ccRCC immune subtypes in TCGA and ICGC cohorts. (**E**) TMB and number of mutated genes in C1/C2 ccRCC patients. (**F**, **G**) NAL and CIN score of C1/C2 ccRCC patients. (**H**, **I**) Expression of ICP genes in C1/C2 ccRCC patients from TCGA cohort and ICGC cohort separately. (**J**, **K**) Expression of ICD factors in C1/C2 ccRCC patients from TCGA cohort and ICGC cohort separately. (**L**, **M**) Expression of NMD factors in C1/C2 ccRCC patients from TCGA cohort and ICGC cohort separately. ^*^padj < 0.05; ^**^padj < 0.01; ^***^padj < 0.001.

Immunotyping has been proven to be of clinical relevance in multiple tumors; it can present tumor-infiltrating immune cells and reveal the ccRCC microenvironment, helping in the identification of suitable patients to receive vaccines. The expression profiles of 577 immune-related genes were screened using the ImmPort database for consensus clustering. ccRCC patients from TCGA were clustered into two immune subtypes (C1 and C2) based on the clustering of immune-related genes (stabilized when K = 2) and their cumulative distribution function (CDF) and function delta area (Figure 4A; Supplementary Figure 3A–3C). Immune-related genes of C1/C2 could be clustered into five types as shown in the heatmap (Figure 4B). A better prognosis had a significant association with C1 (Figure 4C) and differed from that in the ICGC cohort (Figure 4D). Consistently, subtype was distributed along variable tumor grades and stages, which indicates that the C1 subtype showed a high proportion of stage I or grade 2/3 patients, whereas C2 showed a higher proportion of stage III/IV or grade 3/4 patients. ccRCC patients were divided into four molecular subtypes (KIRC1-4) according to TCGA cohort molecular classification. Interestingly, C1 accounted for the majority of KIRC1/2 subtypes, while C2 accounted for most KIRC3/4 subtypes. Subtype distribution across patients diagnosed with differential TNM pathologies was irregularly clustered; 11% of N1 patients belonged to C2, which was nearly four times the percent of patients belonging to C1 (3%) (Supplementary Figure 3D). In summary, immunotyping is a possible prognostic marker for ccRCC patients and has a superior accuracy to the usual grading and staging, which was consistent across different cohorts.

Overexpression of *AHNAK2* and loss of *VHL* and *CDKN2A* are correlated with poor prognosis in patients with ccRCC [24, 25], and are generally used as prognostic markers for ccRCC. We analyzed the differences in the expression of *AHNAK2*, *CDKN2A*, and *VHL* between the C1 and C2 immunotypes in TCGA and ICGC cohorts. The results of TCGA cohort showed that AHNAK2 was significantly overexpressed in the C2 subtype, but the expression of *VHL* and *CDKN2A* was not significantly different (Supplementary Figure 3E). However, the results of the ICGC cohort did not agree with those of TCGA cohort, indicating that these prognostic markers may not be suitable as potential antigens due to their inconsistent expression profiles (Supplementary Figure 3F). Thus, immunotyping is superior to *VHL*, *AHNAK2*, and *CDKN2A* expression profile in prognosis prediction of ccRCC patients.

### Molecular characteristics of immune subtypes

TMB is a clinically related parameter associated with immunotherapy response. We detected somatic mutations and CNV in TCGA cohort between the two immune subtypes. TMB of the C2 subtype was higher than that of C1, and a higher number of mutant genes was present in C2 (Figure 4E; Supplementary Figure 3G). Among all tested genes, including *VHL* and *PBRM1*, the frequency of missense mutations was the highest in both immune subtypes. Mutation in *PBRM1* plays a suppressing role in ccRCC [26] and occurs at a lower frequency in the C2 subtype (37%) than in the C1 subtype (44%), indicating that C2 may be more sensitive to immunotherapy (Supplementary Figure 3H, 3I). However, no significant dissimilarity in CNV between C1 and C2 was observed (Supplementary Figure 3J). A recent study reported that ccRCC tumors show a high infiltration of CD8^+^ T cells, favorable *PBRM1* mutation depletion, and enrichment for unfavorable chromosomal losses of 9p21.3 [27].

Similarly, the results of CNV analysis showed that CNV loss occurred frequently in chromosome 9 of the patients in the C2 subtype (Supplementary Figure 4A). Neoantigen load (NAL) is a predictive biomarker for ICI therapy. The higher NAL in C2 subtype patients suggests a higher immunogenicity of the mRNA vaccine in this subtype (Figure 4F). Chromosomal instability is the main cause of tumor evolution, and tumor cells that show chromosomal instability opt for chronically active innate immunity pathways to metastasize to remote organs [28]. Hence, ccRCC patients with the C2 subtype have a higher risk of metastasis (Figure 4G). Homologous recombination deficiency (HRD) can lead to sensitivity to poly (ADP-ribose) polymerase inhibitors and is used as a biomarker for monitoring treatment efficacy [29]. MRNAsi is an index that can be used for quantification of stemlike indices and has a significant correlation with patient outcomes, and high mRNAsi is associated with upregulation of immunosuppressive checkpoints [30]. Unfortunately, we could not determine the difference in HRD and mRNAsi between the two subtypes (Supplementary Figure 4B, 4C).

We detected the AS of 901 potential neoantigens between the two subtypes. PSI difference was noted in 337 potential neoantigens (37.4%) between the two subtypes. The immune system induces diverse repertoire of antigen receptors, which are expressed by B and T cells and are capable of recognizing a variety of protein antigens [31]. Downregulated expression ICP-related genes can sensitize cells to immunotherapy, and immunogenic cell death (ICD) can stimulate the dysfunctional anti-tumor immune system. In TCGA cohort, significant and differential expression of 32 ICP genes were found between the two subtypes (Figure 4H), and most of them had a higher expression in the C2 subtype.

We also verified 10 ICP genes that had differential expression between the two subtypes in the ICGC cohort (Figure 4I). A total of 29 ICD factors and four NMD factors were significantly and differentially expressed between the two subtypes from TCGA cohort (Figure 4J–4L), and five ICD factors and four NMD factors were verified in the ICGC cohort (Figure 4K–4M). Therefore, reflection of the expression levels of ICP and ICD modulators can be achieved through immunotyping, which is potentially a therapeutic biomarker for mRNA vaccines.

### Cellular characteristics of immune subtypes

**Figure 5:**
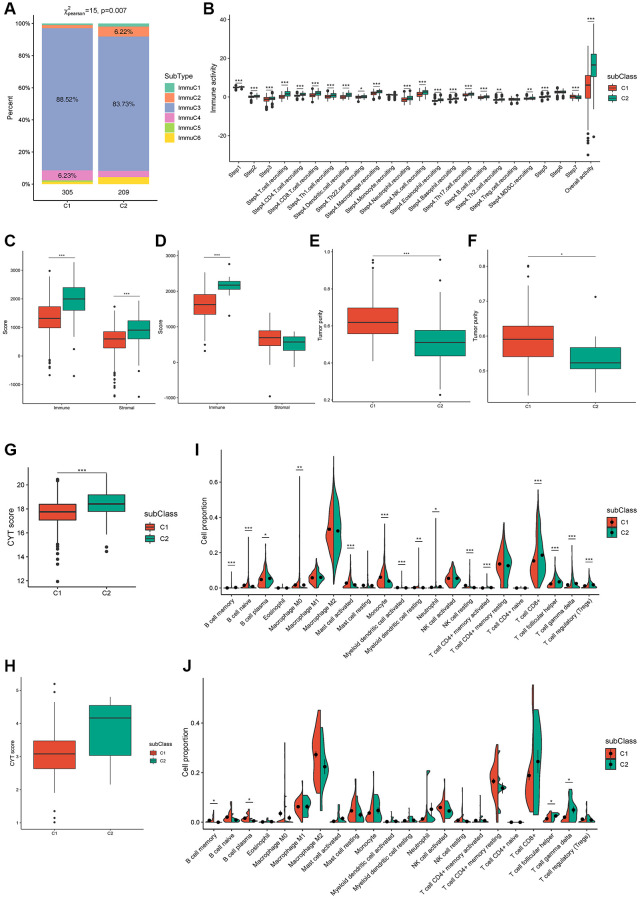
**Cellular characteristics of immune subtypes.** (**A**) Distribution of immune subtypes (ImmuC1-C6) across C1/C2 ccRCC patients. (**B**) Overall activity of immune cells in C1/C2 ccRCC patients. (**C**, **D**) Immune score and stromal score of C1/C2 ccRCC patients from TCGA and ICGC cohorts. (**E**–**H**) Tumor purity and CYT score of C1/C2 ccRCC patients from TCGA (**E**, **G**) and ICGC (**F**, **H**) cohorts. (**I**, **J**) Immune cell proportions of C1/C2 ccRCC patients from TCGA and ICGC cohorts separately. ^*^padj < 0.05; ^**^padj < 0.01; ^***^padj < 0.001.

To demonstrate immunotyping reliability, we analyzed the percentage of six earlier demonstrated pan-cancer immune subtypes (ImmuC1-ImmuC6) among the C1 and C2 immunotypes. The proportions of pan-cancer immuC3 were 88.52% and 83.73% in the C1 and C2 immunotypes, respectively, and immuC4 was the secondary subtype (6.23%) in the C1 immunotype, whereas immuC2 was the secondary subtype (6.22%) in the C2 immunotype. Compared to the C1 immunotype, the lack of immuC5 and abundance of immuC6 in the C2 immunotype may induce some differences between them (Figure 5A). These outcomes correspond to the higher survival observed in the C1 immunotype tumors than in the C2 immunotype tumors. Tumor immunophenotype systematic tracking is required for understanding cancer immunity fundamental mechanisms and improvement of cancer immunotherapy clinical benefits [32]. As shown in Figure 5B, the overall activity of immune cells in the C2 group was significantly higher than that in the C1 group, and most anti-cancer immune response steps correlated with this observation. In particular, the difference in Th2 cell-recruiting activity during the trafficking of immune cells to tumors was consistent with other reports, which showed that the increase in the expression of type-2 T helper cell signature was associated with low survival in ccRCC, papillary RCC, and chromophobe RCC [24].

In patients, immune and stromal scores can help determine tumor purity and immune cell infiltration in the tumor microenvironment, with high immune scores indicating cytolytic good immune responses and better prognosis [33, 34]. Consistently, immune and stromal scores were remarkably higher in the C2 subtype in TCGA cohort (Figure 5C) and the immune scores of the two subtypes were verified in the ICGC cohort (Figure 5D). In addition, the tumor purity of C2 was remarkably lower than that of C1 in both TCGA and ICGC cohorts (Figure 5E, 5F). CYT reflects the cell-killing function and can be used to assess immune-mediated attacks against cancer cells; moreover, it is associated with the mutational burden [35]. The CYT score from TCGA and ICGC cohorts indicated a higher mutational burden and more complex immune microenvironment in the C2 subtype (Figure 5G, 5H). The proportion of 15 types of immune cells, comprising memory B cells, naïve B cells, plasma B cells, M0 macrophages, activated mast cells, monocytes, activated myeloid dendritic cells, resting myeloid dendritic cells, neutrophils, resting natural killer calls (NKCs), activated CD4+ memory T cells, CD8+ T cells, follicular helper T cells, gamma delta T cells, and regulatory T cells (Tregs) was remarkably different between the two subtypes in TCGA cohort (Figure 5I). Although memory B cells, plasma B cells, follicular helper T cells, and gamma delta T cells were verified in the ICGC cohort, some other immune cell types, such as CD8+ T cells, resting NKCs, and activated CD4+ memory T cells showed the same trend (Figure 5J). These results indicated that patients with the C2 subtype had a significant tumor infiltration of CD8+ T cells, [27] suggesting that these patients were more suitable for our mRNA vaccine. Taken together, these results suggest that the immune subtypes are promising candidates for mRNA vaccines, and patients with C2 tumors with/without an immunosuppressive microenvironment are potentially more suitable for mRNA vaccination.

### ccRCC immune landscape

**Figure 6:**
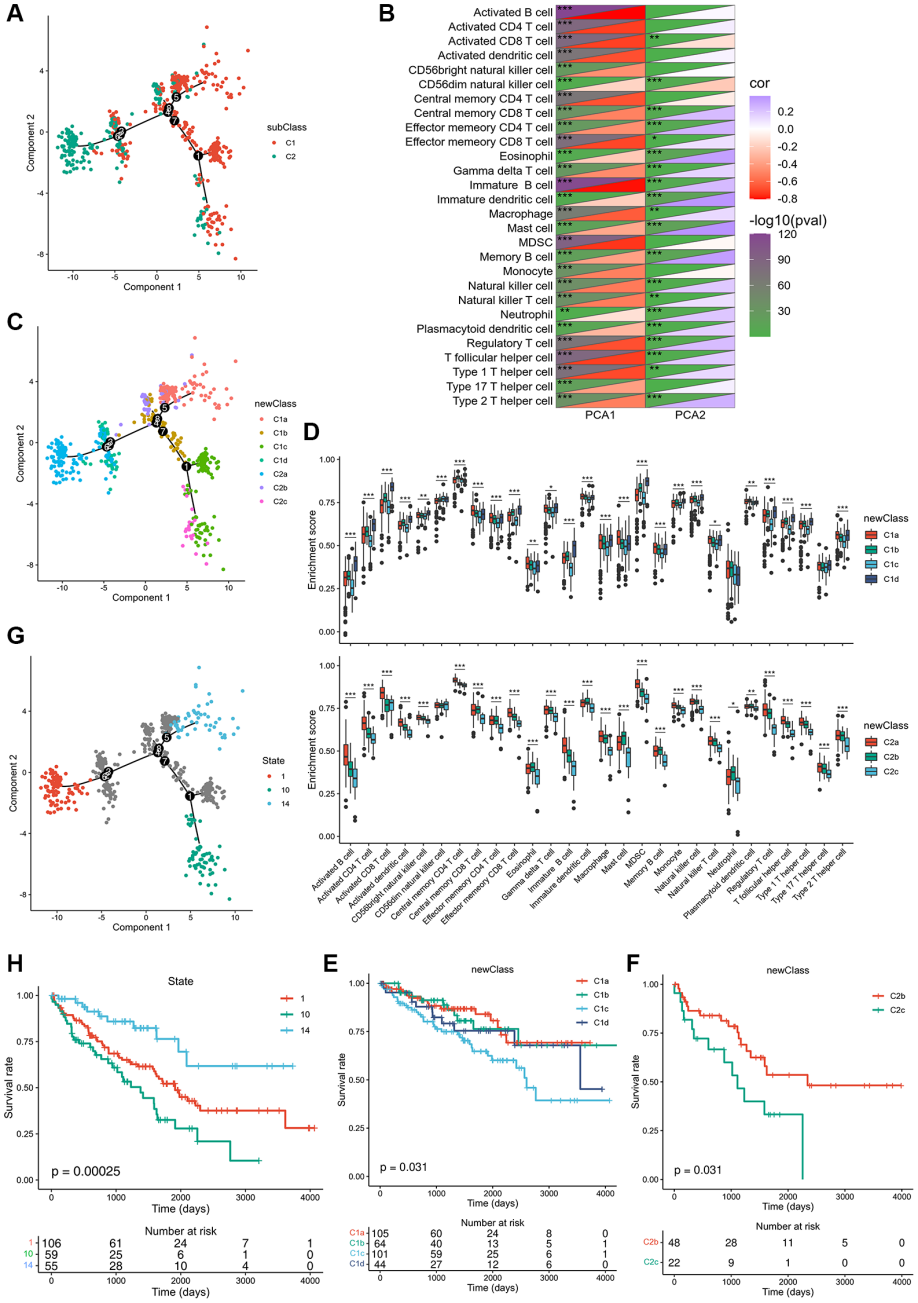
**Immune landscape of ccRCC.** (**A**) Immune landscape of ccRCC. (**B**) Heat map of two principal components with 28 immune cell signatures. (**C**) Immune landscape of the subsets of ccRCC immune subtypes. (**D**) Differential enrichment scores of 28 immune cell signatures in the above subsets. (**E**, **F**) The prognostic status of each subset of C1 and C2 separately. (**G**) Immune landscape of samples from three extreme locations and (**H**) their prognostic status. ^*^padj < 0.05; ^**^padj < 0.01; ^***^padj < 0.001.

The immune landscape of ccRCC was created from 28 immune cell types of pan-cancer gene expression profiles using ssGSEA and monocle (Figure 6A). C1 integral distribution was opposite to that of C2. The correlation between PCA1/2 and 28 types of pan-cancer immune cells is shown in Figure 6B. The pan-cancer immune cells did not show any positive correlation with PCA1 expression. Nevertheless, activated and immature B cells, myeloid-derived suppressor cells, and T follicular helper cells were the most negatively correlated immune cells. Immature dendritic cells, mast cells, and memory B cells showed the highest positive correlation with PCA2. Moreover, differential distribution was displayed in the same subtype, indicating significant intra-cluster heterogeneity within the subtypes. Based on the location of immune cell populations, C1 was further divided into four subsets defined as C1a, C1b, C1c, and C1d, and C2 was further divided into three subsets separately defined as C2a, C2b, and C2c (Figure 6C). The enrichment score of several immune cells showed significant difference between every subset (Figure 6D). C1c and C2c showed lower enrichment scores for most immune cells, such as activated B cells, activated CD4 T cells, activated CD8 T cells, central memory CD8 T cells, effector memory CD4 T cells, effector memory CD8 T cells, and memory B cells, suggesting that patients with these subsets may be more suitable for mRNA vaccination. C1a subtype patients showed the highest survival rates, while subtypes of C1c and C2c showed the worst survival rates (Figure 6E, 6F). In addition, samples that showed utmost immune landscape distribution positions were compared in terms of prognosis, and the probability of survival was the best in a group 14 of patients, consistent with the aforementioned results (Figure 6G, 6H). Taken together, the immune landscape based on immune subtypes can be used for identification of immune components of each ccRCC patient and prediction of their prognoses and can preferably be used to provide a personalized mRNA vaccine therapy.

### ccRCC hub genes and gene co-expression module identification

**Figure 7:**
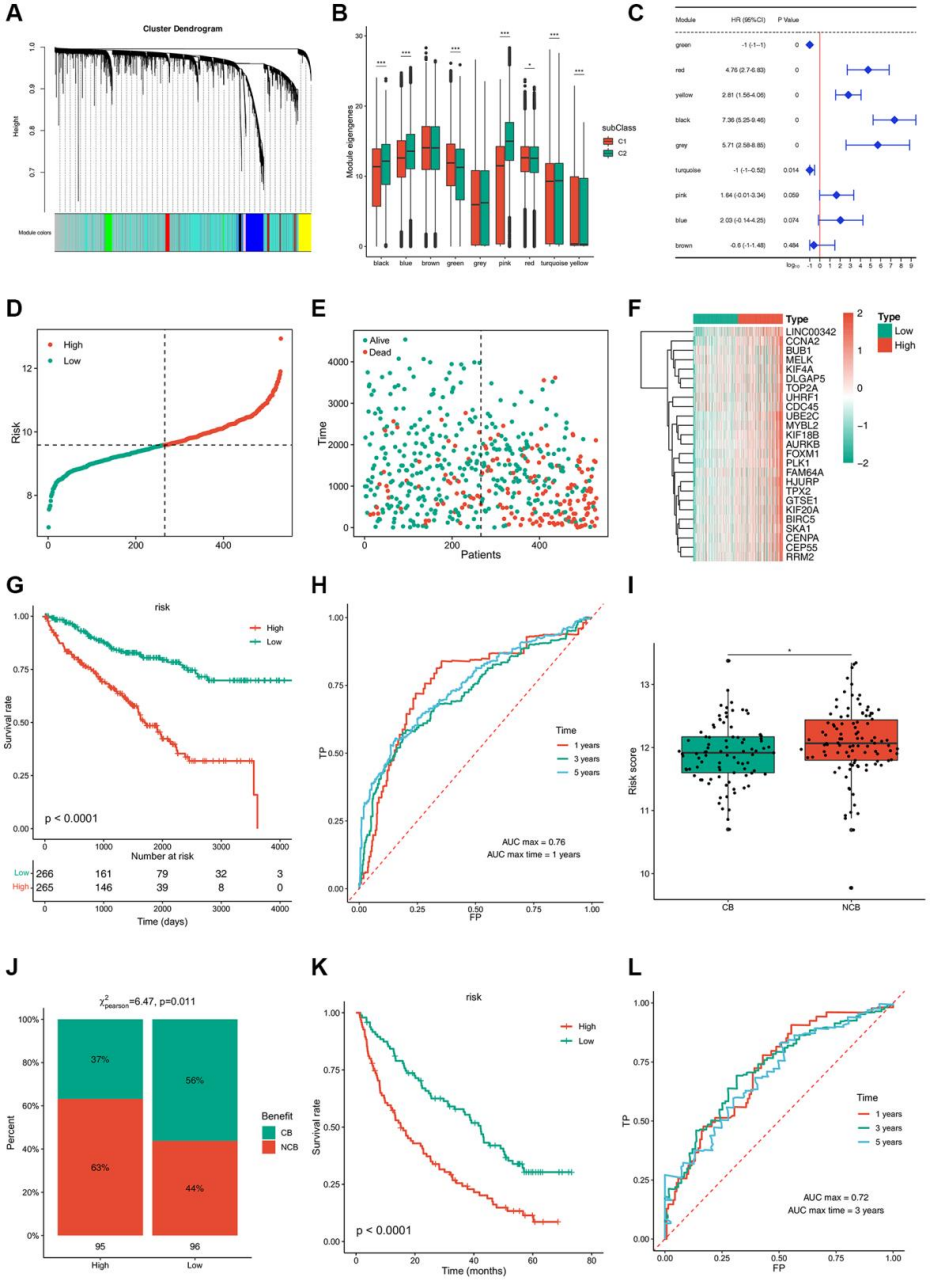
**Immune gene co-expression modules and immune hub genes for ccRCC identification and the established risk-estimating model.** (**A**) Dendrogram of all differentially expressed genes clustered based on a dissimilarity measure (1-TOM). Clustering of samples. (**B**) Differential distribution of feature vectors of each module in ccRCC subtypes. (**C**) Forest maps of single-factor survival analysis of nine modules of ccRCC. (**D**–**F**) Risk-estimating model was established from prognostic modules and (**G**) their prognostic status from TCGA cohort. (**H**) ROC curve of risk of ccRCC patients from TCGA cohort. (**I**, **J**) Risk score of ccRCC patients treated with PD-1 and (**K**) their prognostic status from TCGA cohort. (**L**) ROC curve of risk of ccRCC patients treated with PD-1 from TCGA cohort. ^*^padj < 0.05; ^**^padj < 0.01; ^***^padj < 0.001.

TCGA co-expression module identification was performed by sample clustering based on WGCNA (Supplementary Figure 5A) with a soft threshold of 4 for a scale-free network (Supplementary Figure 5B, 5C). Nine co-expression modules obtained from TCGA are shown in black, blue, green, pink, red, turquoise, yellow, gray, and brown (Figure 7A; Supplementary Figure 5D). The expression of the black, blue, green, pink, red, turquoise, and yellow module eigengenes was significantly different between the two subtypes. C2 showed higher eigengenes in the black, blue, pink, and turquoise modules and lower eigengenes in the green, red, and yellow modules (Figure 7B). Furthermore, the black, gray, red, and yellow modules showed significant positive correlations with prognosis, whereas the green and turquoise modules were notably negatively correlated with prognosis (Figure 7C). GO enrichment of the green, black, gray, and turquoise modules indicated that the prognostic modules were related to small molecule catabolic processes, nuclear division, organelle fission, and epidermis development. Regulation of membrane potential, kidney epithelium development, and renal tubule development were enriched in the KEGG pathway, indicating that the prognostic modules were closely linked to kidney health (Supplementary Figure 5E).

A risk estimation model was established to predict tumor risk according to the expression of 25 hub genes that were obtained from prognostic modules (Figure 7D–7F). Low-risk patients showed a better survival rate (Figure 7G). The time-dependent receiver operating characteristic (ROC) curve suggested that this model can accurately predict the 1- to 5-year survival rate of ccRCC patients (Figure 7H). Thus, this model can be used to identify patients who can benefit from mRNA vaccines through risk scoring and provide a reference for immunotherapy. Next, 592 patients treated with programmed cell death protein 1 (PD-1) blockade were divided into clinical benefit (CB) and no clinical benefit (NCB) groups; NCB patients showed a higher risk score (Figure 7I). In contrast, 56% of patients with CB were concentrated in the low-risk group, while 63% of patients in the high-risk group had a poor survival rate (Figure 7J, 7K). The time-dependent ROC curve suggested that this model can accurately predict 1- to 5-year survival rate of ccRCC patients treated with PD-1 blockade (Figure 7L). Collectively, these results provide evidence for the accuracy of the risk prediction model, based on which, more patients can benefit from mRNA vaccines.

## DISCUSSION

The study aimed to identify potential tumor antigens and immune subtypes of ccRCC for mRNA vaccine development. We constructed the aberrant AS and mutational landscape of ccRCC and identified *ARHGEF3* as a promising mRNA vaccine candidate. *RhoGEFs* play critical roles in various signaling cascades and cellular processes and are involved in multiple cancers. *ARHGEF3* downregulation may be associated with invasion, metastasis, and proliferation in osteosarcoma because this gene specifically activates *RHOA* and *RHOB*, which play a role in bone cell biology [36, 37]. According to the Human Protein Atlas database, high *ARHGEF3* expression is favorable for patient survival and potentially a prognostic marker for renal cancer, endometrial cancer, and head and neck cancer, and others. In contrast, elevated *ARHGEF3* expression is a crucial contributor to the pathogenesis of nasopharyngeal carcinoma (NPC) by inhibiting cell apoptosis and potentially used as a novel marker for prognosis and an effective target for treatment [38]. Alternatively, spliced transcript variants encoding different isoforms have been found for *ARHGEF3*, suggesting that aberrant splicing isoforms are responsible for the diverse effects of *ARHGEF3* in various tumors. According to TCGA SpliceSeq database, the PSI of the alternate promoter from exon 7 in ccRCC patients was twice as high as normal. Hence, the mRNA vaccine of *ARHGEF3* for ccRCC patients was designed to target this specific splicing isoform.

The aberrant splicing transcript of *ARHGEF3* not only had an association with bad prognosis but also with higher APCs and infiltration of B cell. Given that the benefit of mRNA vaccines is applicable for some cancer patients only, we divided ccRCC patients into two groups (C1 and C2) according to 577 immune-related genes. The two immune groups showed distinct molecular, cellular, and clinical characteristics. The worse prognosis of patients with the C2 subtype in both ICGC and TCGA cohorts indicates that we can use immunotyping as prognostic marker in patients with ccRCC as it is superior to traditional grading and staging in terms of accuracy. Further, the prognostic performance of immune subtype is superior to that of traditional tumor markers (*VHL*, *AHNAK2*, and *CDKN2A*) in ccRCC patients. As a promising predictor of immune checkpoint inhibitor efficacy, the tumor’s ability to produce new antigens is possibly reflected indirectly by TMB. A high TMB is an indicator supporting the use of monotherapy in children and adults with solid tumors. In addition to its prognostic potential, immunotyping can predict mRNA vaccine therapeutic response. The molecular characteristics of immune subtypes indicated that the TMB, number of mutated genes, NAL, and chromosomal instability in patients with C2 tumors were higher than those in patients with C1 tumors, suggesting that ccRCC patients with the C2 subtype may respond better to mRNA vaccines.

ccRCC poses a challenge to known standards in cancer immunology because of the modest mutation burden; however, it responds to immunotherapies, and a higher CD8+ T cell infiltration has traditionally been linked to poor prognosis [39, 40]. Aberrant splicing transcripts of ARHGEF3 play a critical role in ccRCC progression and development and can be processed directly and presented to CD8+ T cells to elicit an immune response. However, the high expression of partial ICPs, such as *CD200R1*, *CD244*, *CD28*, *CD48*, *CD80*, *CD86*, *ICOS*, *PDCD1LG2*, *TNFRSF9*, and *TNFSF14*, in C2 tumors suggests that the tumor microenvironment is immunosuppressive; thus, mRNA vaccine inhibition may be caused by eliciting an effective immune response. Further, the response to ICIs is related to the depletion of *PBRM1* mutations and enrichment of chromosomal losses of 9p21.3, [27] while the elevated expression of partial ICD modulators, such as *EIF2A*, *IL1B*, *TFAM*, *TLR5*, and *TLR7*, in C2 tumors suggests that mRNA vaccines are effective in these immune subtypes. Moreover, the immunological complexity of the ccRCC landscape considerably suggests heterogeneity between individual cases and within the same immune subtype, thus decreasing the immune components needed to develop personalized mRNA vaccine-based therapeutics. Patients in group 10 showed the worst prognosis, consistent with the results for the C1c and C2c subsets. C1c and C2c showed significantly lower scores for activated CD8 T cells, eosinophils, activated B cells, effector memory CD4 T cells, effector memory CD8 T cells, and monocytes than for other subsets, indicating that C1c and C2c are immunologically “hot,” and therefore, should respond better to mRNA vaccines. Furthermore, the risk estimation model established based on 25 hub genes of prognostic modules can be used to accurately determine CB ccRCC patients and enable more patients to benefit from mRNA vaccines.

In conclusion, ARHGEF3 is a potential ccRCC antigen for mRNA vaccine development. Candidates with immune subtype 2, especially C2c, were the most suitable for vaccination. This study provides a theoretical basis for developing anti-ccRCC mRNA vaccines, predicting patient outcomes, and selecting vaccination candidates. We recommend further study and validation of the identified vaccine antigens and other prognostic markers. Moreover, we are attempting to verify the potential application of ARGEF3 as a promising vaccine antigen. By constructing an aberrant AS *AHRGEF3* cell line, cell-line-derived xenograft, and ccRCC patient-derived tumor xenograft, we intent to understand how aberrant AS *AHRGEF3* induces changes in the tumor immune environment in ccRCC. Moreover, the intrinsic connection between the novel ccRCC antigen *ARHGEF3* and immune subtype warrants further studies. Similar investigations should be conducted for other cancer types as well.

## MATERIALS AND METHODS

### Data acquisition and preprocessing

Percent spliced in (PSI) values for the splice events of all genes and samples of kidney renal clear carcinoma (KIRC) were downloaded from TCGA SpliceSeq database. MuTect2 Variant Aggregation and Masking (*n* = 336) GDC Hub, Phenotype (*n* = 985) GDC Hub, GISTIC - focal score by gene (*n* = 536) GDC Hub, and gene expression RNAseq (*n* = 607) were obtained from the clinical and follow-up information from the XENA TCGA-KIRC database. The clinical characteristics of the samples from TCGA are shown in Supplementary Table 2. The RECA-EU project data were obtained from the ICGC cohort.

### AS and enrichment analysis

The difference in PSI between ccRCC and normal control was determined using the t-test, and significance was set at *p* < 0.05. Gene ontology (GO) enrichment analysis of the target gene set was performed by the ClusterProfiler package [41] in R (version “4.1”).

### Gene expression analysis

Gene expression data (FPKM values) from RNAseq (*n* = 607) of ccRCC and normal control from TCGA-KIRC database were compared using the limma package in R (version “4.1”) [42] and filtered using |LogFC| > 1 and *p* < 0.01 as threshold criteria.

### Cox proportional-hazards model and survival analysis

The Cox proportional-hazards model and survival analysis were used for regression modeling of the characteristic gene and OS or disease-free survival (DFS) data using the coxph function in the survival package [43] in R (version “4.1”) and filters with *p* < 0.05 as the threshold. The effect sizes were displayed in a forest plot using corresponding modeling parameters. For multifactor Cox analysis, multigene pairs were built using the coxph function of the survival package for regression modeling. The genes identified in the survival analysis were grouped after screening for the median expression level, and the OS was fitted using the survfit function of the survival package. Analysis and visualization were performed using the ggsurvplot function of the Survminer package in R (version “4.1”).

### TIMER2 analysis

Tumor Immune Estimation Resource [42] was employed for analysis and visualization of the association between tumor-infiltrating cells (TICs) and ccRCC-related genes using analytical modules for somatic mutations, gene expression, clinical outcomes, and somatic copy number alterations. Adjustment of purity was performed by Spearman’s correlation analysis. Statistical significance was set at *p* < 0.05.

### Consensus clustering analysis

Screening of the immune gene set downloaded from ImPort using single-factor Cox yielded 577 immune-related genes associated with prognosis. The prognosis-related immunogenetic expression data of TCGA-KIRC patients were screened and normalized with the median, and then analysed using the R package ConsensusClusterPlus for Consensus clustering. The clustering algorithm was k-means algorithm, and sampling was performed 500 times.

### Analysis of somatic mutation and copy number variation (CNV) between immune subtypes

We downloaded TCGA-KIRC somatic mutation maf file from the TCGA, calculated the TMB using the maftools package in R, and visualized the mutation type. The frequency of CNV gain or loss was calculated through GISTIC file from the Xena database, visualized using maftools.

### Analysis of intertype immune activity

Data for the immune activity of TCGA-KIRC were downloaded from the TIP database, and the t-test was conducted for testing differences in the immune activity scores of different subtypes.

### Homologous recombination defects, new antigen loads, chromosomal instability, and dryness index analysis

The DNA damage repair (DDR) score was downloaded from https://gdc.cancer.gov/about-data/publications/PanCan-DDR-2018 and The Cancer Immunome Atlas (TCIA) database. Maftools was used to calculate the new antigen load TMB from TCGA-KIRC. A list of unstable chromosomal genes was obtained from a published paper [44], and the sum of the expression values of these genes was used as the chromosomal instability score. mRNAsi was used to calculate the dryness index of tumor subtypes according to a previously published paper [PMID:29625051], and *t*-test was used to calculate the differences between the four indicators.

### Immune cell analysis

The immune, matrix, and tumor purity evaluations of different TCGA-KIRC subtypes were performed using R-package estimate, and the expression means of the genes granzyme A and perforin 1 were used as immune cytolytic activity (CYT) scores. The immuno-immersion score file from TCGA was downloaded from the TIMER2 database, the data related to TCGA-KIRC sample was included, the CIBERSORT score data were analyzed, the immune immersion score of test data set was calculated using CIBERSORT, and a Wilcox-test/t-test was used to calculate the differences in the immunization score index.

### Immune landscape of ccRCC

Data of 28 pancellular immune cell types were downloaded from a published paper, [45] using the sSGSEA algorithm to analyze the relative abundance of each immune cell type in TCGA-KIRC. The DDRTree dimension reduction of TCGA-KIRC was then carried out using the monocle package, and the correlation between the first two main components and 28 pancellular immune cell types was calculated. The subtype was subdivided according to the pseudotime analysis of TCGA-KIRC samples. The relative abundance difference of 28 pancellular immune cell types in each subcategory was determined using single-factor analysis of variance (ANOVA).

### WGCNA co-expression network construction

The expression level of differentially expressed genes in TCGA-KIRC was screened, and the co-expression network of TCGA-KIRC expression matrix was constructed using the WGCNA package. We set 0.8 as the threshold for the average connectivity of the scale-free fit index and network with the change of the soft threshold parameter, when soft threshold (softpower) changed from 1 to 10. The softpower was set to 4, such that it met the scale-less network, a co-expression network was built, and the prognosis module was identified, wherein the minimum number of genes of the module was 30 and the combined correlation was greater than 0.7. The Pearson correlation coefficient between the genes in the module and the module feature genes was calculated. |cor| > 0.9 and *p* < 0.001 was used to identify hub genes, and a risk score was determined according to a multivariate Cox model of the hub genes.

### Data availability

The data supporting the findings of this study are available from the corresponding author upon reasonable request.

## Supplementary Materials

Supplementary Figures

Supplementary Table 1

Supplementary Table 2
