# Supplementary material for: Identification of neoantigens and immunological subtypes in clear cell renal cell carcinoma for mRNA vaccine development and patient selection
Source: Aging (Albany NY). 2023 Jun 13;15(11):5190–214. doi: 10.18632/aging.204798 (PMC10292886; doi:10.18632/aging.204798)
Supplement: Supplementary Figures [file aging-15-204798-s001.pdf]

SUPPLEMENTARY FIGURES

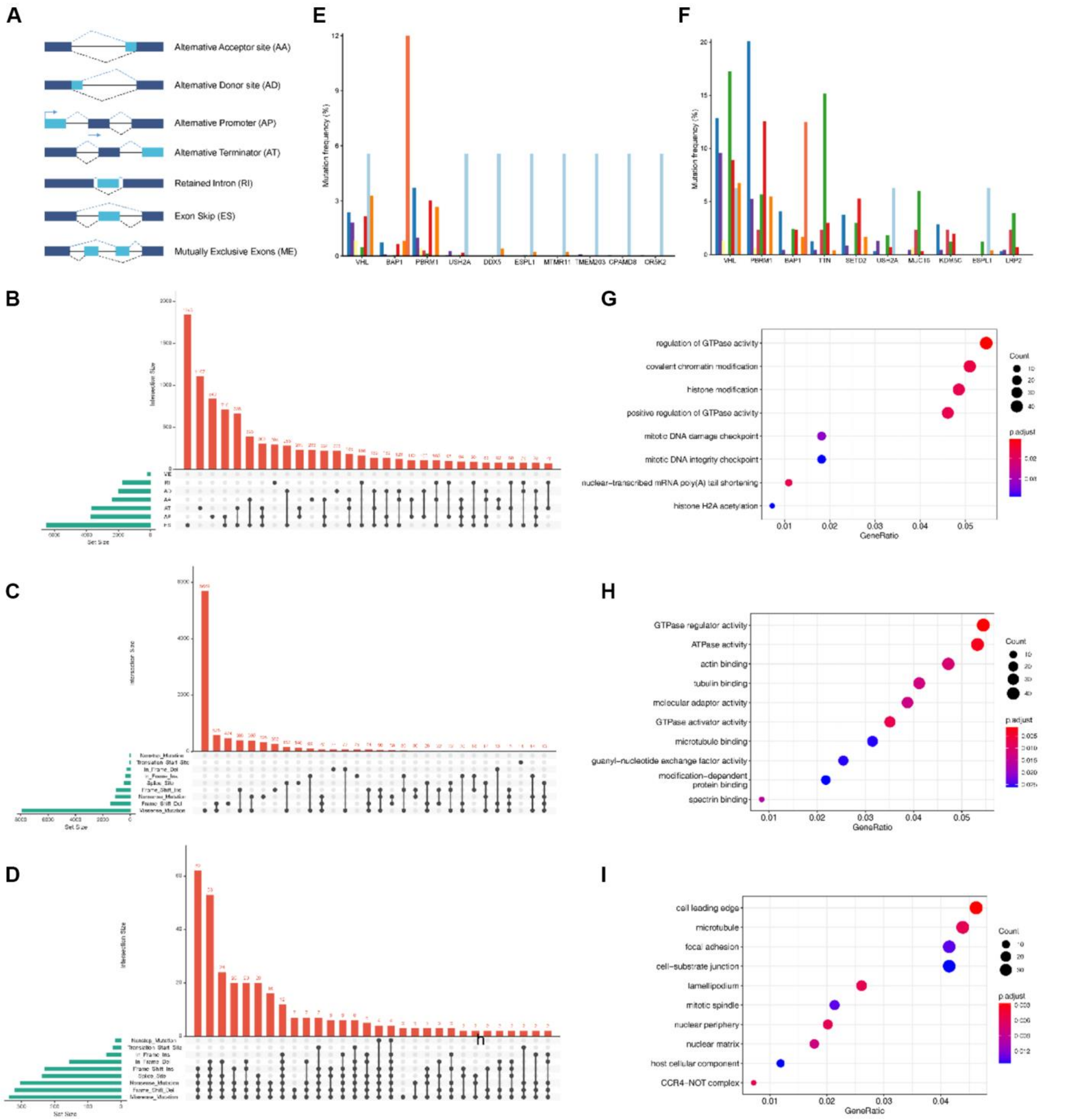

Supplementary Figure 1. Potential antigen identification and enrichment of gene ontology (GO). (**A**) Seven AS events in ccRCC patients. (**B**) Number of genes involved in AS events. (**C**, **D**) Frequency of somatic mutations and genes/patients involved. (**E**, **F**) The mutation frequency of top 10 genes in all samples/ccRCC patients. (**G**–**I**) biological process (BP), molecular function (MF), and cellular component (CC) enrichment of 901 potential antigens.

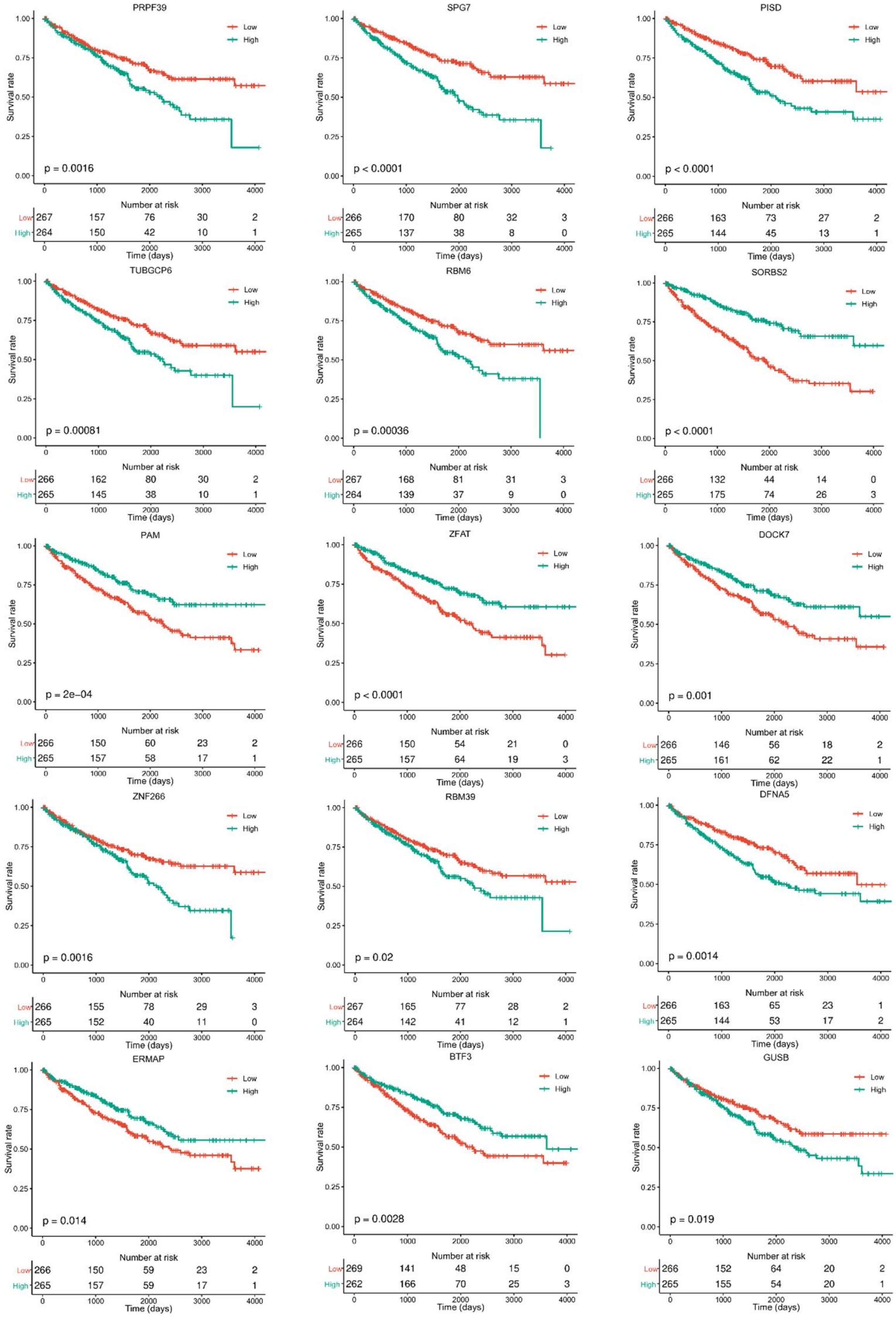

Supplementary Figure 2. Fifteen candidates significantly associated with the overall survival rate. Effect of PRPF39, SPG7, PISD, TUBGCP6, RBM6, SORBS2, PAM, ZFAT, DOCK7, ZNF266, RBM39, DFNA5, ERMAP, BTF3, and GUSB on overall survival rate.

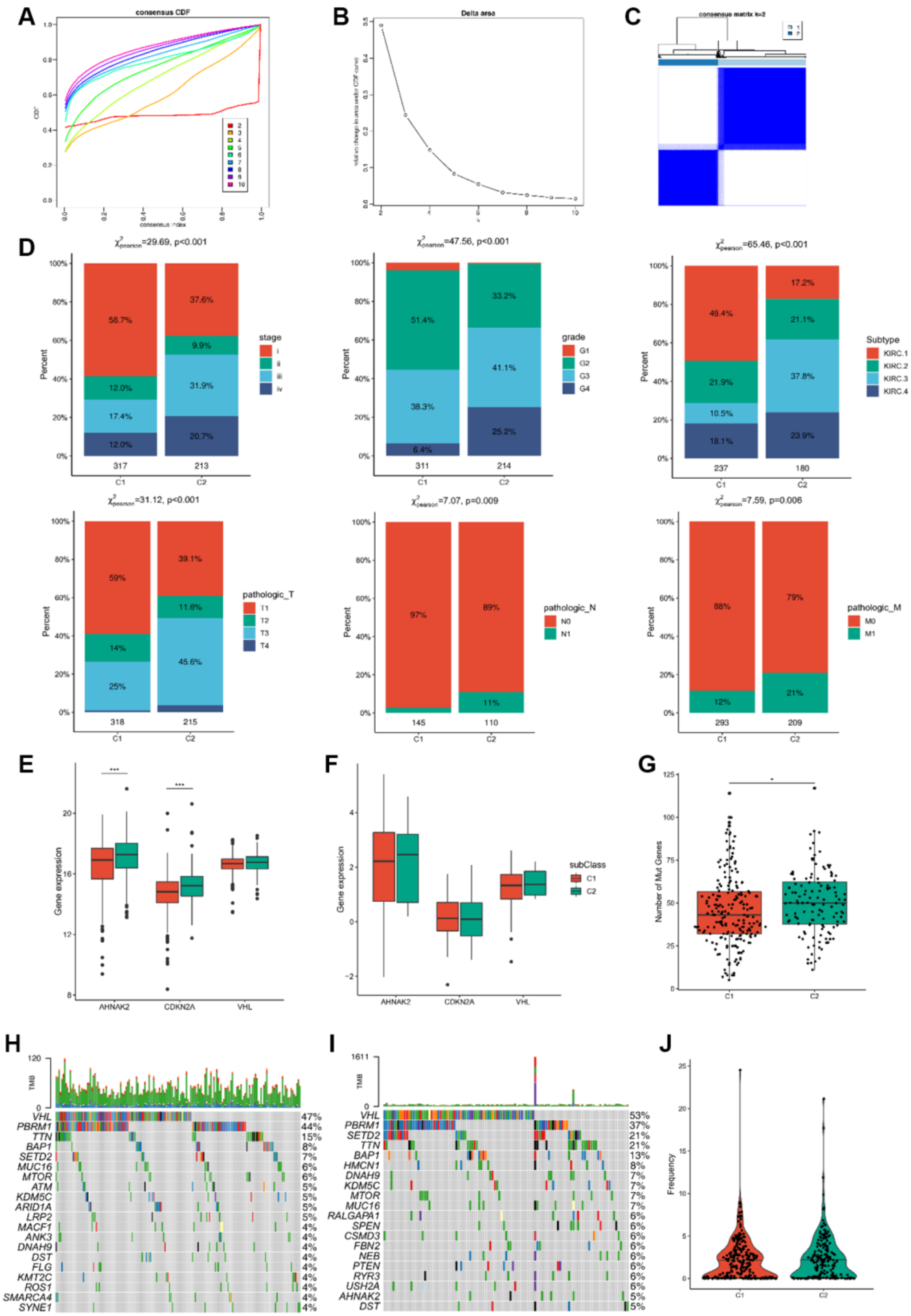

Supplementary Figure 3. Identification of potential immune subtypes of ccRCC patients. (**A**) Cumulative distribution function curve and (**B**) delta area of immune-related genes in TCGA cohort. (**C**) Sample clustering heat map. (**D**) Distribution of C1/C2 across ccRCC stages, grades, molecular subtypes, T, N, M staging of TCGA cohort. (**E**, **F**) The expression of ccRCC patient biomarkers in C1/C2 subtypes. (**G**) Number of mutated genes in C1/C2 ccRCC patients. (**H**, **I**). Twenty highly mutated genes in C1/C2 ccRCC patients. (**J**) CNV frequency of C1/C2 ccRCC patients.

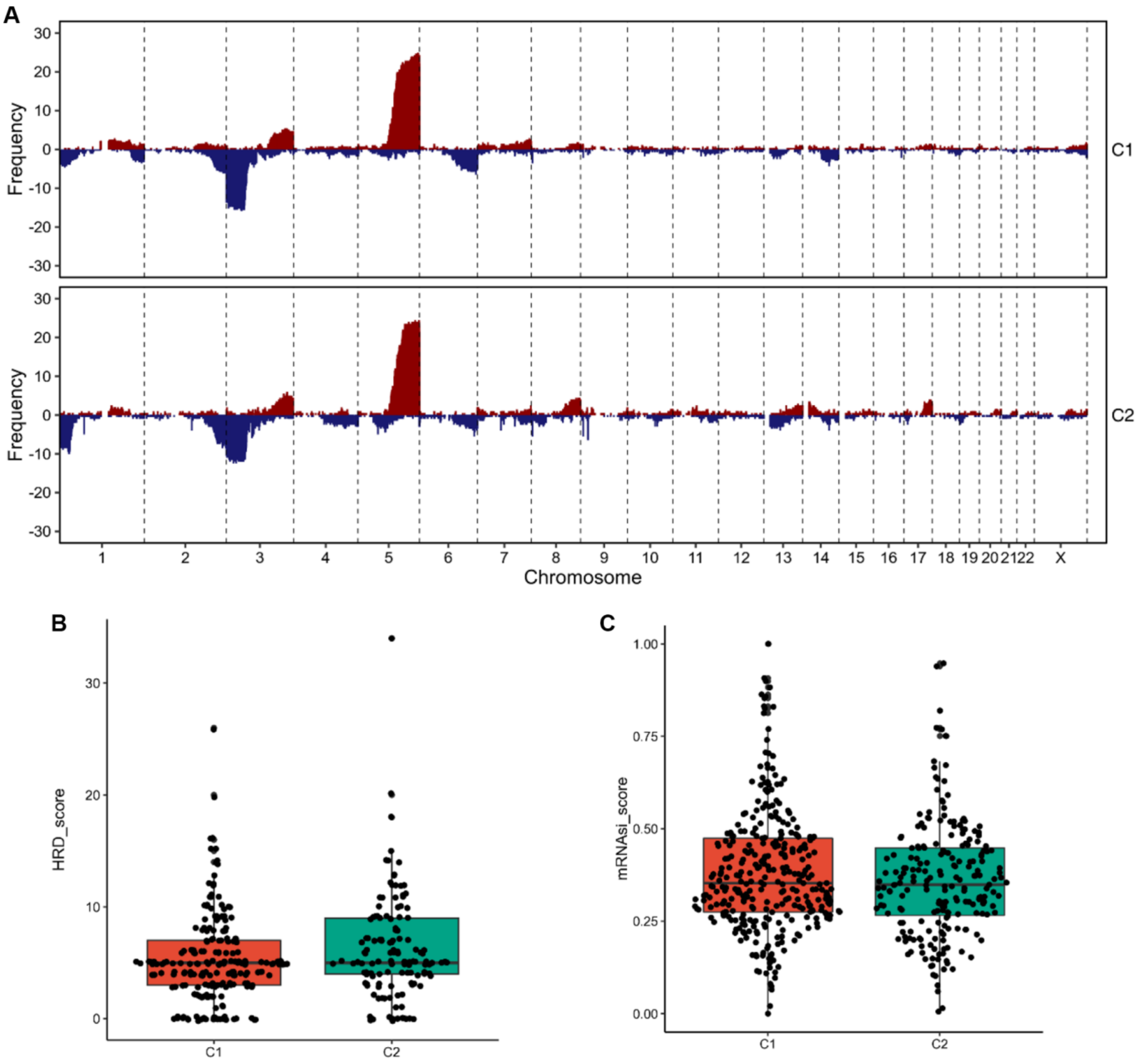

Supplementary Figure 4. Molecular characteristics of immune subtypes. (**A**) Overview of CNV in C1/C2 ccRCC patients. (**B**, **C**) HRD score and mRNAsi score of C1/C2 ccRCC patients. **p*adj < 0.05; ***p*adj < 0.01; ****p*adj < 0.001.

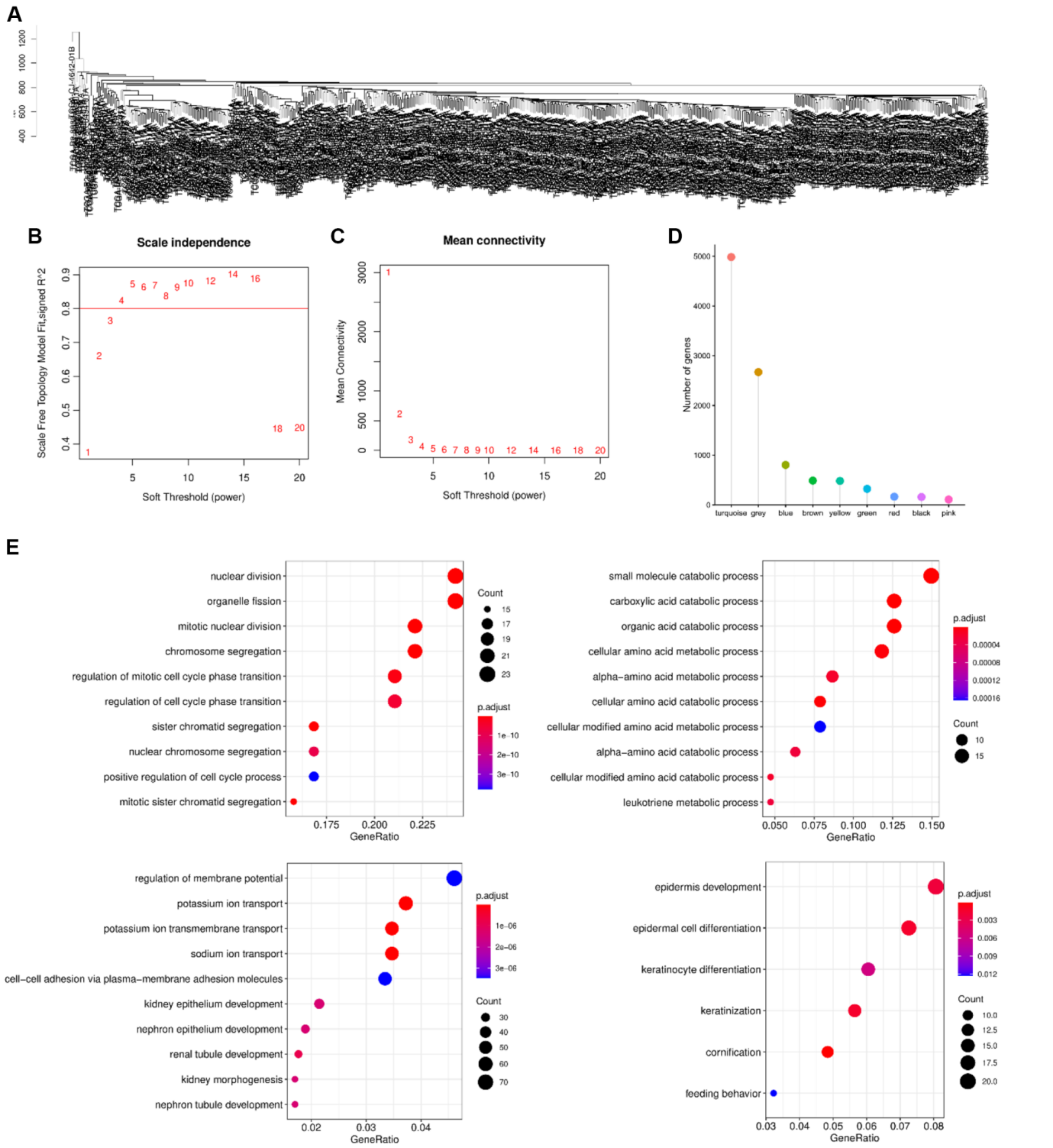

Supplementary Figure 5. Immune gene co-expression modules and immune hub genes for ccRCC identification. (**A**) Clustering of samples. (**B**) Scale-free fit index for various soft-thresholding powers (β). (**C**) Mean connectivity for various soft-thresholding powers. (**D**) Gene numbers in each module. (**E**) BP, MF, CC, and KEGG enrichment of green, black, gray, and turquoise modules. The dot size and color intensity represent the gene count and enrichment level, respectively.
