# Supplementary material for: Identification of neoantigens and immunological subtypes in clear cell renal cell carcinoma for mRNA vaccine development and patient selection
Source: Aging (Albany NY). 2023 Jun 13;15(11):5190–214. doi: 10.18632/aging.204798 (PMC10292886; doi:10.18632/aging.204798)
Supplement: Supplementary Table 2 [file aging-15-204798-s003.pdf]

Supplementary Table 2. The clinical characteristics of samples from TCGA.

Characteristic	Tumor	Normal
**Sample**	531	71
Stage I	265	−
II	58	−
III	123	−
IV	82	−
Grade I	13	−
II	230	−
III	206	−
IV	74	−
KIRC subtype 1	147	−
2	90	−
3	93	−
4	86	−
Pathologic T1	271	−
T2	70	−
T3	179	−
T4	11	−
Pathologic N0	239	−
N1	16	−
Pathologic M0	422	−
M1	78	−
